# Biocontrol of the oriental armyworm, *Mythimna separata*, by the tachinid fly *Exorista civilis* is synergized by Cry1Ab protoxin

**DOI:** 10.1038/srep26873

**Published:** 2016-06-21

**Authors:** Xingfu Jiang, Lei Zhang, Haixia Yang, Thomas W. Sappington, Yunxia Cheng, Li zhi Luo

**Affiliations:** 1 State Key Laboratory for Biology of Plant Diseases and Insect Pests, Institute of Plant Protection, Chinese Academy of Agricultural Sciences, Beijing, 100193, China; 2 College of Plant Science and Technology, Huazhong Agricultural University, Wuhan 430070, People’s Republic of China; 3 USDA-ARS Corn Insects & Crop Genetics Research Unit, Genetics Laboratory, Department of Entomology, Iowa State University, Ames, IA 50011, USA

## Abstract

Tritrophic interactions between *Mythimna separata*, its tachinid parasite *Exorista civilis* and the Cry1Ab were examined. Although 6th instar *M. separata* mortality increased with increasing Cry1Ab concentration, some tolerance was evident. Likewise, parasitization by *E. civilis* resulted in only 18% host mortality. However, combination of Cry1Ab and *E. civilis* parasitization resulted in a significant Cry1Ab dose-dependent increase in mortality over that of either alone, including a 50–56% synergistic increase in efficacy at the two concentrations tested. Pupal weight, adult emergence and lifetime fecundity of *M. separata* derived from larvae surviving both agents were negatively affected. The ability of *E. civilis* to parasitize and subsequently develop on the host was not adversely influenced by Cry1Ab. Instead, pupation rate increased significantly among host larvae fed 3.125 μg/g Cry1Ab diet. Overall, our results demonstrate that use of Cry1Ab to control *M. separata* not only is compatible with the use of the tachinid parasitoid, but that the two methods can act synergistically to manage this destructive pest, provide support for the safety of transgenic Cry1Ab Bt plants in China. This example of two independent pest management strategies acting synergistically against a difficult pest offers a new perspective of broad significance in striving for agricultural sustainability.

The oriental armyworm, *Mythimna separata* (Lepidoptera: Noctuidae), a typical long-distance migratory insect, is a major, polyphagous pest of grain crops in China and other Asian countries, causing huge crop production and economic losses nationwide annually^1^,^2^,^3^,^4^. From 1950 to 2013, the average annual area of cropland in China infested by *M. separata* was 5.28 million ha^5^. With the recent adjustment in agricultural planting structure in China, maize has become the most extensively planted food crop nationwide, increasing from 29 million ha in 2007 to 35 million ha in 2011. Consequently, maize has become the most important host plant of *M. separata* in China^5^,^6^, and infestations in the north and northeast in 2012 accounted for a 2.9% yield loss in total maize production^5^,^7^.

Transgenic crops producing toxins from *Bacillus thuringiensis* (Bt) are widely used and have proved highly effective in the management of insect pests in many countries^8^. In China, transgenic Bt cotton expressing the Cry1Ac protoxin has been commercially planted since 1997. It is effective against certain lepidopteran pests and improves biocontrol by beneficial insects^9^,^10^. For the sake of successful and sustainable management of maize insect pests in China, including *M. separata,* insect resistant transgenic Bt maize expressing Cry1Ab recently was approved for small scale planting in the field for purposes of ecological safety evaluation. Previous studies documented the influence of Bt crops expressing Cry1Ab on larval development and survival of *M. separata*, but the results were not consistent and even contradictory^11^,^12^,^13^. The conflicting results are probably attributable to the Bt and non-Bt varieties used for larval food in the experiments, because different concentrations of Cry1Ab toxin are expressed in different varieties or in the same varieties during different growth stages^14^,^15^,^16^. Although *M. separata* is not the primary target pest of current transgenic maize hybrids, it is at least somewhat susceptible to the Cry1Ab toxin^11^,^12^. It and its natural enemies are inevitably exposed to Cry1Ab maize owing to *M. separata* preference for maize as a host plant^6^. Therefore, research on the effects of Cry1Ab on *M. separata* and its tritrophic effects on *M. separata*’s natural enemies is important in evaluating Bt maize ecological safety, as well as in building a foundation for a biological control method for this pest.

Two issues are intertwined in evaluating simultaneous control of a pest by a Bt toxin and a natural enemy: biological effects of the toxin on the natural enemy, and net efficacy. Interactive effects of the two agents on biological characteristics (e.g., development rate, reproductive potential, longevity, acute mortality, growth, behavior) of both the host pest and the parasitoid may be individually positive, negative, or neutral, and may differ depending on life stage^17^,^18^,^19^. The net effect of these interactions on efficacy of the combined agents against the pest can be antagonistic, additive (independent), or synergistic^20^. Exposure of a parasitoid to the transgenic Bt toxin will be primarily tritrophic through the target herbivore host that has ingested the toxin. Observed host-insect mediated, or tritrophic, effects of Bt proteins on parasitoid biology are often neutral^21^,^22^,^23^. Although negative tritrophic effects are sometimes reported, most are attributable to indirect effects of Bt-induced host death or poor host quality rather than via direct toxicity^21^,^22^,^23^,^24^,^25^,^26^,^27^,^28^. Positive tritrophic affects on the parasitoid are possible^19^,^29^, but probably tend to arise indirectly through compromise of host immunity or increased development time after exposure to the toxin^17^,^30^. Reciprocally, host defenses compromised by a parasitoid may make the host more vulnerable to the Bt toxin^18^. Most studies of tritrophic effects of Bt on parasitoids have focused on wasps, whereas such studies on tachinid fly parasitoids are largely lacking.

We previously examined dose-dependent effects of Bt toxins (Cry1Ac or Cry1Ab) on growth and development of *M. separata* and its natural parasitoid wasp, *Microplitis pallidipes*^31^,^32^, as well as associated physiological and molecular insecticidal mechanisms^33^,^34^. However, dose-dependent effects of Cry1Ab on survival, growth and reproduction of *Exorista civilis*, a crucial tachinid parasitoid in the field^35^,^36^, and on *M. separata* when the latter is exposed to both agents simultaneously, remain unknown. Here we have addressed this knowledge gap by evaluating *M. separata* survival, growth and development, and lifetime fecundity when exposed to different concentrations of Cry1Ab in artificial diet and to *E. civilis* parasitism, alone and in combination. We also examined the effect of host-mediated exposure to Cry1Ab on *E. civilis* biology and parasitism. We report the novel finding of synergistic efficacy of Cry1Ab and *E. civilis* on *M. separata* mortality. Furthermore, for the Cry1Ab doses tested against *M. separata*, no negative tritrophic effects on *E. civilis* were observed. In addition to the importance of our results for *M. separata* management and biosafety of *E. civilis* in Bt crops, the demonstration of synergistic control of a serious pest by a classical biological control agent in concert with a transgenic Bt toxin opens new horizons for developing novel strategies for pest management.

## Results

### Mortality of host *M. separata* larvae exposed to combinations of Cry1Ab and parasitism

**Figure 1:**
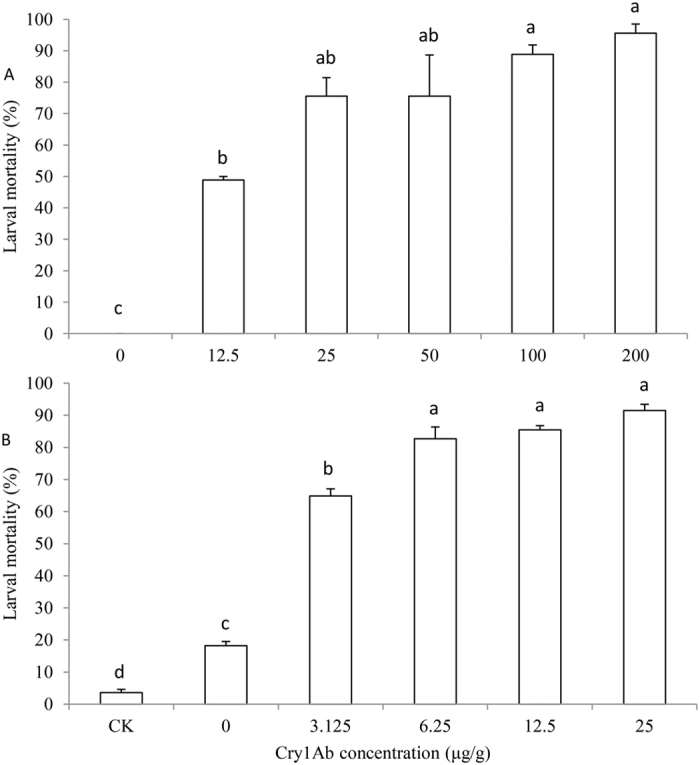
Mortality of 6th instar *M. separata* fed on artificial diet containing different concentrations of Cry1Ab when unparasitized (**A**) or parasitized (**B**) by the tachinid fly *E. civilis*. Larvae comprising the CK group in panel B were not exposed to either Cry1Ab or parasitism. Data are presented as mean ± SEM. Bars sharing the same letter are not significantly different at 5% level by Tukey’s HSD test. Sample sizes of each treatment in panel A are 90. Sample sizes in panel B are 168, 229, 167, 150, 170 and 189 from left to right, respectively.

Mortality of non-parasitized 6th (last) instar *M. separata* was significantly affected by concentration of Cry1Ab in the diet (*F*_5, 12_ = 37.226, *P* < 0.0001), ranging from 48.9% to 95.6% at the higher concentrations tested (12.5 μg/g to 200 μg/g) (Fig. 1A). Mortality in all of the Cry1Ab treatments was significantly greater than that of the control group, but mortality at the four highest concentrations did not differ significantly from one another (Fig. 1A). *E. civilis* parasitism alone without Cry1Ab treatment resulted in 18.2% host larval mortality, which was significantly higher than mortality of the unparasitized control (Fig. 1B). When parasitized by *E. civilis* and simultaneously exposed across a range of lower Cry1Ab concentrations (3.125 μg/g to 25 μg/g), 6th instar *M. separata* mortality was significantly affected, ranging from 64.8% to 91.5% (Fig. 1B). The lowest concentration of Cry1Ab tested (3.125 μg/g) caused significantly higher mortality of parasitized *M. separata* compared to Cry1Ab-free diet (Fig. 1B). Probit analysis indicated a LC_50_ of 11.243 μg/g Cry1Ab in artificial diet for non-parasitized 6th instar larvae, versus only 1.863 μg/g Cry1Ab when parasitized by *E. civilis* (Table 1).

The procedure of Johnson and Gould^20^ confirmed that the effect of *E. civilis* on mortality of *M. separata* 6th instars fed Cry1Ab-impregnated diet was synergistic at the two concentrations tested. The effect of synergism was 56.3% in the 12.5 μg/g Cry1Ab diet treatment, and 50.1% in the 25 μg/g treatment.

### Effects of combined exposure to Cry1Ab and parasitization on *M. separata* pupation and reproduction

Among 6th instar *M. separata* that survived exposure to both Cry1Ab and tachinid parasitism, pupation rate (*x*^2^ = 26.479, *df* = 4, *P* < 0.0001) and pupal weight (*F*_4, 108_ = 24.201, *P* < 0.0001) were significantly affected by protoxin concentration (Table 2). Both parameters decreased significantly with increasing Cry1Ab concentrations. No *M. separata* pupae survived to emergence when they had been both parasitized by *E. civilis* and exposed to the highest concentration of Cry1Ab tested (25 μg/g) as 6th instars. Protoxin concentration treatments did not significantly affect pupal duration (F_3, 78_ = 0.992, *P* = 0.401). *M. separata* adult emergence rate (*x*^2^ = 14.858, *df* = 3, *P* = 0.002) was significantly affected by protoxin concentration, showing significant decrease as increasing of Cry1Ab concentrations. Pre-oviposition period (*F*_3, 50_ = 20.260, *P* < 0.0001), and lifetime number of eggs laid per female (*F*_3, 40_ = 15.305, *P* < 0.0001) were significantly affected by *E. civilis* parasitism. Although there were numerical trends of increasing adult pre-oviposition period and decreasing lifetime fecundity among parasitized larvae with increasing Cry1Ab concentrations, the differences were not significantly different from those of parasitized larvae on control (non-Cry1Ab) diet (Table 2).

It is possible that reduced pupal size caused by larval rearing diet containing different concentrations of Cry1Ab led to the observed adult reduction in lifetime fecundity of *M. separata*. To examine this possibility, adult lifetime number of eggs laid was regressed on pupal weight, revealing a significant positive linear relationship (y = 3.77 × −208.15, R^2^ = 0.56, *F*_1, 42_ = 52.37, *P* < 0.0001).

### Host-mediated effects of Cry1Ab delivered via artificial diet on *E. civilis*

**Figure 2:**
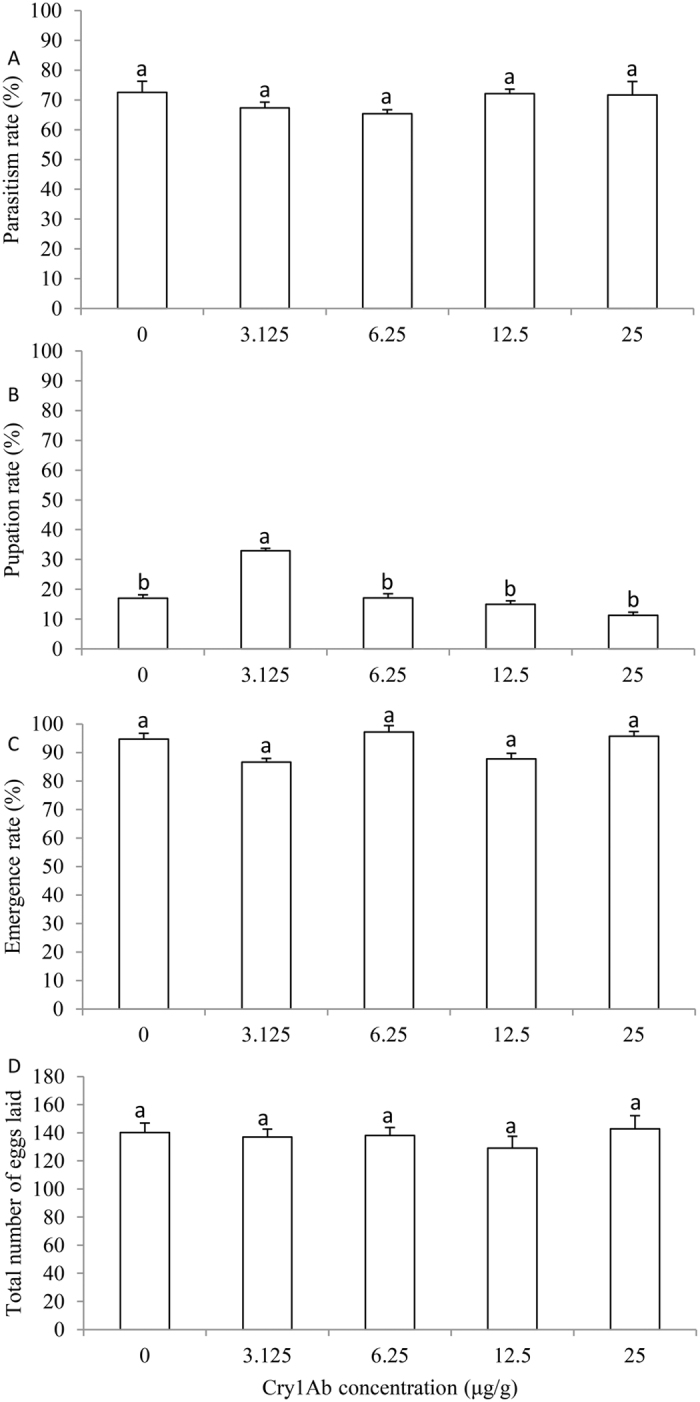
Influence of Cry1Ab concentrations in the host (*M. separata*) larval diet on parasitism rate (**A**), pupation rate (**B**), emergence rate (**C**) and life time fecundity (**D**) of the tachinid parasitoid, *E. civilis*. Data are presented as mean ± SEM. Bars sharing the same letter are not significantly different at 5% level by Tukey’s HSD test. Sample sizes (left to right) for each treatment are 51, 49, 46, 50 and 42 (**A**); 229, 182, 240, 273 and 418 (**B**); 39, 60, 36, 41 and 46 (**C**); and 17, 22, 15, 14, and 14 (**D**), respectively.

*E. civilis* parasitism rate (*F*_4, 20_ = 3.353, *P* = 0.054), adult emergence rate (*F*_4, 10_ = 1.894, *P* = 0.151), and lifetime number of eggs laid per female (*F*_4, 80_ = 0.297, *P* = 0.897) were not significantly influenced by feeding of the host larva for two days on diet containing different concentrations of the Cry1Ab protoxin (Fig. 2A,C,D). In contrast, *E. civilis* pupation rate (*F*_4, 10_ = 90.168, *P* = 0.023) was significantly enhanced when host larvae fed on diet containing the lowest concentration of Cry1Ab tested (3.125 μg/g) compared to the non-Cry1Ab control and the remaining Cry1Ab concentrations, which did not differ from each other (Fig. 2B).

## Discussion

Assessing the safety of Bt crops toward non-target pest and beneficial insects continues to receive much attention, and is one of the rate-limiting steps in the commercialization of Bt crops in China and other countries. Impacts of a Bt crop on non-target pests differ depending on insect pest species and Bt toxin. Several studies have linked outbreaks of secondary non-target pests to fields planted with transgenic crops. For example, population abundance or crop damage caused by mirid bugs^37^,^38^, beet armyworm, *Spodoptera exigua*^39^,^40^, and aphids^41^ tend to increase in Bt cotton fields. However, the increased density and damage of mirids in transgenic fields is largely attributable to reduced pesticide use against the target insect pest^38^,^42^. *S. exigua* has only low susceptibility to Cry1Ac protoxin expressed in Bt cotton, and a sublethal dose ingested by the larva triggers increased long-distance flight activity in the adult. This response may improve the chances of escaping adverse local conditions before oviposition, while amplifying the area damaged by outbreak immigrant populations^43^. A few non-target insect pests have developed resistance to Bt toxins^44^,^45^.

In China the main target pest in Bt maize is the Asian corn borer, *Ostrinia furnicalis*. Earlier studies suggested at least some efficacy of Cry1Ab against *M. separata* as well^11^,^12^, but results were equivocal about whether it would be enough to provide good control by itself. In our experiments, although *M. separata* larvae showed increased mortality with increasing Cry1Ab concentration, the LC_50_ of 11.243 μg/g is relatively high. The concentration of toxin in fresh leaf tissue of most Cry1Ab Bt maize hybrids ranges from 1 to 17 μg/g^28^. Furthermore, no significant increase in mortality was observed beyond 25 μg/g (Fig. 1A), suggesting last instar *M. separata* is fairly tolerant to the Cry1Ab toxin, consistent with the findings of Yun *et al*.^11^ regarding older larvae^11^. Hence, other management measures compatible with the partial efficacy of Cry1Ab will be necessary to control *M. separata* once Bt maize is commercialized in China. This system is representative of many similar situations worldwide, where there is a need to manage an array of non-target pests attacking a Bt crop without reverting to a broad-spectrum insecticide. The increasing demands of meeting global food security needs in environmentally sustainable ways drive a growing urgency to explore new tactics and strategies for suppressing certain pest species in combination with existing technologies that primarily target other pests.

Although natural enemies can be important assets for managing most insect pests in the field, their often low lethality and slow activity seldom provide adequate control by themselves. *E. civilis* is the main natural enemy of *M. separata* in maize, but host larval mortality is usually low because of *M. separata*’s relatively high tolerance to this parasitoid. However, when we combined *E. civilis* and Cry1Ab in the laboratory against *M. separata*, synergistic effects on efficacy were observed. In our experiments, the LC_50_ of Cry1Ab for parasitized 6th instars was 6-fold less than for non-parasitized larvae. In the absence of parasitism by *E. civilis*, Cry1Ab doses of 12.5 and 25 μg/g caused 48.9% and 75.6% mortality of 6th instars respectively, while parasitism alone resulted in 18.2% mortality. However, when *E. civilis* and Cry1Ab were combined, they acted synergistically to generate mortalities of 85.4% and 91.5% at the two doses tested. Finally, even among the larvae of *M. separata* that survived the combination of Cry1Ab and *E. civilis* parasitism, subsequent pupal size, adult emergence and lifetime fecundity were negatively influenced. The results suggest that a combination of Cry1Ab and *E. civilis* applied to control late instars of *M. separata* may be an effective management measure for this destructive insect pest.

A few studies have detected negative tritrophic effects of Bt crops on natural enemies, but in almost all cases these are not due to direct effects of the toxin on the beneficial insects. Instead, a decrease in natural enemy population density may simply reflect a decrease in the host pest population density, or compromised host-quality caused by the Bt toxin can result in poorer performance of the host’s parasitoids or predators^25^,^27^. A lack of direct negative tritrophic effects of Bt crops on pest natural enemies is strikingly illustrated when the latter are provided hosts or prey from strains resistant to the Bt toxin that have developed normally on a Bt host plant^22^,^23^,^46^,^47^. In our study, the capacity of *E. civilis* to emerge as adults, develop a normal lifetime complement of eggs, and locate and parasitize host larvae were not adversely affected by being reared in a naturally tolerant host fed on Cry1Ab-containing diet. Size of host larvae was relatively uniform in the experiment, and lack of difference in *E. civilis* parasitism rate suggests larval host activity was not influenced by its exposure as a 6th instar to the different concentrations of Cry1Ab used in this study^48^,^49^,^50^. Furthermore, *E. civilis* survivorship was not negatively influenced by Cry1Ab, even at the highest concentrations tested. Indeed, its pupation rate increased significantly on host larvae fed diet containing the lowest concentration (3.125 μg/g) of Cry1Ab. After surviving the intoxicated host larva, subsequent adult eclosion rate and lifetime egg production of *E. civilis* also were not adversely affected. Our results are the first demonstration that host-mediated Cry1Ab toxin is not obviously harmful to a tachinid parasitoid. Similar results of no deleterious tritrophic effects from transgenic Bt plants have been reported for a number of parasitoid wasp species^21^,^23^,^26^.

An important limitation of our study is that exposure of *M. separata* larvae was restricted to the 6th instar, the larval stage most tolerant to pesticides, including Bt. This decision was dictated by the need to ensure adequate numbers of surviving larvae on which to test *E. civilis*, which only attacks 6th instars. Therefore, the experimental design does not precisely mimic field conditions in which most of the 6th instars present in Bt maize will have survived much longer exposure to the toxin than in our study. There are several implications of this design which must be borne in mind when interpreting the results. Our dose-response data apply directly only to previously unexposed 6th instars; the LC_50_s we observed may be lower or higher than what would be observed for 6th instars exposed to the toxin throughout the larval stage. Our measure of 6th instar mortality caused by Cry1Ab alone is probably less than the expected cumulative mortality for larvae exposed through all instars. Tritrophic effects on parasitoids might have been evident if reared on larvae surviving a longer exposure to the toxin, especially if lifetime exposure reduces 6th instar host quality compared to the previously naive 6th instars used in our study. There are many possible scenarios of exposure and potential outcomes which will require future laboratory and field experimentation to address.

Nevertheless, our results are of value in demonstrating synergism between Cry1Ab and *E. civilis* against 6th instar *M. separata* under the conditions of our experiment, and they carry a number of important ramifications. We hypothesize that planting Cry1Ab Bt maize in areas where *E. civilis* is common may provide good control of *M. separata*, whereas reliance on either agent alone will prove inadequate. Older larvae, which are more tolerant of Bt toxins than younger larvae, are quite mobile, and movement from non-Bt fields into Bt fields may be fairly common. This situation is true of many other lepidopteran pests as well. Escape of young larvae from exposure to the toxin followed by exposure of mature larve to sub-lethal doses can promote evolution of resistance. The lack of evidence for negative tritrophic influence of Cry1Ab toxin on this generalist parasitoid suggests that augmentative releases of laboratory reared *E. civilis* could help manage insect pests that are naturally tolerant or that have evolved resistance to transgenic Cry1Ab plants. Such a strategy could potentially delay Bt resistance development as well. Thus, the results of our evaluation of tritrophic effects of Bt toxin on *M. separata* and its main tachinid parasitoid not only provide strong support for the safety of transgenic Bt maize expressing Cry1Ab in China. Perhaps more importantly, they open new prospects for considering coordinated management of difficult pests by synergizing classical biocontrol tactics with deployment of transgenic Bt crops.

The synergism we observed between Cry1Ab and *E. civilis* against *M. separata* raises the fascinating question of the physiological and molecular mechanisms responsible. We hypothesize that the Bt protein depresses the host immune response and defensive enzyme activity, making it more difficult to protect the host against invasion of the hemocoel by the parasitoid. Our previous work demonstrated that haemocyte number and the activities of detoxification enzymes and proteases decreased significantly after exposure of *M. separata* larvae to low concentrations of Cry1Ac toxin^33^. The balance among superoxide dismutase, catalase and peroxidase in the larval midgut also was disturbed significantly^33^. In addition, a molecular Cry protein receptor (*MsCAD1*) has been characterized from *M. separata*, which binds the Cry1Ab toxin affecting toxicity^34^. It is also possible that the immune response to *E. civilis* larvae diverts resources, or otherwise compromises, host capacity to detoxify Cry1Ab which is continuously being ingested. Future study of the mechanisms by which the two agents work together to overcome the powerful host immune system promises rich rewards in understanding this system and insect immunity in general.

## Materials and Methods

### Insects

*M. separata* used in the experiments were from a colony that originated from a population collected in Kangbao county Hebei province (41.87°N, 114.6°E). The colony had been maintained for more than 10 generations when the experiments began. Larvae were reared individually in a 12-well plate (wells 3 cm dia. × 2 cm deep) on artificial diet comprised of soybean flour, yeast powder, casein, dry maize leaf powder, cholesterol, sugar, agar, ascorbic acid, sorbic acid, methyl p-hydroxybenzoate, vitamin solution and distilled water, developed for *M. separata* by our research group (patent ZL201010197333.X)^51^. Rearing conditions included a constant temperature of 23 ± 1°C, approximately 70% RH and photoperiod of 14L:10D (light period beginning 07:00 local time). Pupae were transferred on the fifth day and adult males and females were allowed to emerge in separate containers. Adults were transferred in pairs to 850-mL jars provided with wax paper for oviposition, and eggs were collected daily. Jars were covered with gauze to facilitate ventilation and adults were provided with 5% honey solution (v/v) replaced daily^31^,^52^.

*E. civilis* were also collected from Kangbao county Hebei province and maintained in the laboratory for 10 generations by rearing on *M. separata*, a common host of *E. civilis* in China. The adults were paired and maintained in a plastic box (26cm × 15 cm × 18 cm) provided with 10% honey solution (v/v) replaced daily.

### Cry1Ab protoxin and diet preparation

Cry1Ab protoxin produced from *Bacillus thuringiensis* Kurstaki (purity >98%) was purchased from Envirologix Inc. (USA). The protein showed as a clear band at about 130 kDa in SDS-PAGE. The purified Cry1Ab protoxin was lyophilized and stored at −70 °C, and dissolved in Na_2_CO_3_ buffer (0.1 mM, pH = 10.0) just before use. During diet preparation, serially diluted Cry1Ab protoxin of the desired concentrations were added to the liquid diet before solidification, and mixed in a blender for about 60 s.

### Mortality of *M. separata* larvae exposed to both Cry1Ab and *E. civilis*

Newly hatched larvae (≤12 h after hatch) of *M. separata* were reared individually in wells of a 12-well plate on normal diet with no Cry1Ab until the first day of the 6th (last) instar. Each 6th instar larva was transferred into another 6-well plate on diet containing Cry1Ab concentrations of 0 (control), 12.5, 25, 50, 100, or 200 μg/g, where they remained until death or pupation. The sixth stadium is about 5 d on normal diet and about 9 d on Bt toxin-impregnated diet. There were 30 larvae per treatment per experiment, and the experiment was replicated three times for a net total of 90 larvae per treatment.

Another group of 6th instar *M. separata* were transferred into a glass box containing mated *E. civilis*. Eggs of this parasitoid are oviposited on the cuticle of the host and are easily visible by eye. When a larva became parasitized once (i.e., one egg only per larva), it was transferred into another 6-well plate and fed on diet containing Cry1Ab at concentrations of 0 (protoxin-free control), 3.125, 6.25, 12.5 and 25 μg/g. This range of concentrations was chosen based on preliminary experiments indicating they would result in about 20–90% mortality of *M. separata* in the presence of *E. civilis*. An additional control of unparasitized larvae reared on artificial diet without Cry1Ab protoxin was included. Larvae were held thus until death or pupation. There were approximately 60 larvae per treatment per experiment, and the experiment was replicated three times.

Larval mortality was recorded daily from the first day of the 6th instar to pupation. Larvae were considered dead if they were unable to move in a coordinated manner when prodded with a blunt probe.

### Pupation and reproduction of *M. separata* surviving larval exposure to combinations of Cry1Ab and *E. civilis*

Pupation rate, 2-day-old pupal weight and pupal duration were recorded for *M. separata* surviving each treatment of Cry1Ab and *E. civilis* larval exposure. Likewise, adults emerging from surviving pupae in each treatment were paired for mating in 850-mL jars provided with wax paper for oviposition. Adult pre-oviposition period, emergence rate and lifetime fecundity were recorded. Samples for recording pupal weight, pupal duration, pre-oviposition period and lifetime fecundity were randomly selected from surviving individuals in each treatment (see Table 2 for sample sizes). Pupae were weighed using an ER182A electric balance (Japan, A&D Co.). Pupal duration was measured in days through successful adult eclosion. Emergence rate was calculated as the number of successfully emerged adults divided by total number of pupae. Pre-oviposition period and lifetime fecundity were determined following the methods employed in previous studies^31^,^52^.

### Host-mediated effects of Cry1Ab on *E. civilis*

We examined the potential for changes in parasitism capacity of *E. civilis* when its host was exposed to Cry1Ab. Sixth instar *M. separata* were placed individually in a 6-well plate on diet containing concentrations of 0, 3.125, 6.25, 12.5 or 25 μg/g Cry1Ab for two days. The larvae were transferred in groups of 10 into a plastic box containing one pair of 5-day-old *E. civilis* for 24 hours. The experiment was replicated five times: i.e., five *E. civilis* females were tested per treatment on a total of about 50 larvae per treatment. Larval parasitism rate was calculated as the presence or absence of eggs laid by *E. civilis.*

We examined whether *E. civilis* population dynamics may be affected by host exposure to Cry1Ab. Sixth instar *M. separata* parasitized only once (i.e., one egg only per larva) were reared in a 6-well plate individually and fed on diet containing concentrations of 0 (control), 3.125, 6.25, 12.5 and 25 μg/g Cry 1Ab until *E. civilis* larvae exited the host and pupated. To guarantee enough surviving *E. civilis* for the experiment, approximately 200 parasitized host larvae were obtained from each of the 0 to 6.25 μg/g treatments, and 300 and 400 were obtained for the higher dose treatments of 12.5 and 25 μg/g Cry 1Ab, respectively. Pupation rate and emergence rate of *E. civilis* were calculated as the number of pupae and adult flies per the total number of host larvae and pupae, respectively. Adults that emerged within each Cry1Ab treatment were paired and provided with 10% honey solution (v/v) as well as five 6th instar host larvae reared on normal diet for oviposition; solution and larvae were replaced daily until *E. civilis* death. Lifetime fecundity per female *E. civilis* was calculated according to the total number of eggs laid per paired female maintained in the plastic box. The experiment was repeated three times.

### Data Analysis

All data obtained from the studies are presented as mean ± SEM. Treatment effects on variables were evaluated by one-way analysis of variance (ANOVA). If the ANOVA indicated a significant difference, the means responsible for that difference were identified using Tukey’s HSD test. All percentage data except host *M. separata* pupation rate and emergence rate were arcsine transformed before ANOVA analysis to meet the assumptions of normality. Differences in host pupation rate and emergence rate between treatments were compared by Chi-square tests. Probit analyses were performed to further compare differences in mortality of host larvae with increasing Cry1Ab concentration in the absence or presence of *E. civilis*. Net efficacy of combined agents was determined to be additive, synergistic, or antagonistic by comparing proportional survival of parasitized and unparasitized larvae on Cry1Ab diet (12.5 and 25 μg/g treatments) to those on normal diet, as described by Johnson and Gould^20^. All statistical procedures were performed with SPSS software (SPSS 17.0).

## Additional Information

**How to cite this article**: Jiang, X. F. *et al*. Biocontrol of the oriental armyworm, *Mythimna separata* by the tachinid fly *Exorista civilis* is synergized by Cry1Ab protoxin. *Sci. Rep.* **6**, 26873; doi: 10.1038/srep26873 (2016).

## Figures and Tables

**Table 1:** Regressions of 6th instar *M. separata* mortality against Cry1Ab concentration in artificial diet, and LC_50_s, when unparasitized or parasitized by *E. civilis.*

**Parasitism**	**Parameters of equation**		Chi-square(χ2)	***P***	LC_50_ and 95% CI (μg/g)
	**Slope**	**intercept**			
Unparasitized	1.310	−1.376	4.405	0.221	11.243 (6.767–15.622)
Parasitized	1.156	−0.312	2.872	0.238	1.863 (0.891–2.813)

**Table 2:** Pupal and reproductive performance of *M. separata* surviving both parasitism by *E. civilis* and consumption of larval diet containing different concentrations of Cry1Ab protoxin.

Cry1Abconcentrations(μg/g)	Pupationrate (%)	Pupal weight(mg)	Pupal period (days)	Emergencerate (%)	Preovipositionperiod (days)	Total no. eggslaid in lifetime
CK	98.1 (162)	310.6 ± 7.3(46) a	11.8 ± 0.2 (54) a	95.0 (159)	5.0 ± 0.2 (20) a	1171 ± 50 (20) a
0	94.1 (187)	252.6 ± 7.8(39) b	11.9 ± 0.2 (54) a	56.3 (176)	6.9 ± 0.3 (17) b	790 ± 39 (17)b
3.125	84.8 (59)	219.4 ± 7.9(26) b	12.2 ± 0.3 (16) a	36.0 (50)	7.5 ± 0.7 (4) b	761 ± 77 (4) b
6.25	84.6 (26)	227.2 ± 6.2(22) b	12.0 ± 0.3 (6) a	27.3 (22)	8.3 ± 0.3 (3) b	734 ± 39 (3) b
12.5	64.0 (25)	180.7 ± 6.3(13) c	11.0 ± 0.3 (6) a	25.0 (16)	No pairs were set up to mate	–
25	68.8 (16)	136.1 ± 7.9(12) d	No pupae survived	–	–	–

CK indicates neither Cry1Ab nor parasitism. Number in the parentheses indicates the corresponding sample size. Data are presented as mean ± SEM. In each column, data sharing the same letter are not significantly different at 5% level by Tukey’s HSD test or Chi-squared tests.
